# Hearing: Travelling Wave or Resonance?

**DOI:** 10.1371/journal.pbio.0020337

**Published:** 2004-10-12

**Authors:** Andrew Bell

## Abstract

A fresh look at classic work of Thomas Gold on how the inner ear processes sound

Sitting in the enveloping quietness of an anechoic chamber, or other quiet spot, you soon become aware that the ear makes its own distinctive sounds. Whistling, buzzing, hissing, perhaps a chiming chorus of many tones—such continuous sounds seem remarkably nonbiological to my perception, more in the realm of the electronic.

Even more remarkable, put a sensitive microphone in the ear canal and you will usually pick up an objective counterpart of that subjective experience. Now known in auditory science as spontaneous otoacoustic emission, the sound registered by the microphone is a clear message that the cochlea uses active processes to detect the phenomenally faint sounds—measured in micropascals—that our ears routinely hear. If the ear were more sensitive, we would need to contend with the sound of air molecules raining upon our eardrums.

What is that process—the mechanical or electrical scheme that Hallowell Davis in 1983 called the ‘cochlear amplifier’ (Davis 1983)—which energises the pea-sized hearing organ buried in the solid bone of our skull?

That question has engaged my curiosity since the late 1970s, when English auditory physicist David Kemp first put a microphone to an ear and discovered the telltale sounds of the cochlea at work (Kemp 1978). Siren-like, the sounds have drawn me into the theory and experiment of cochlear mechanics, now as part of a PhD course at the Australian National University in Canberra. I am studying the micromechanics of this process from a theoretical point of view, and investigating whether a resonance picture of some kind can be applied to the faint but mysterious sounds most cochleas emit.

Kemp's discoveries are rightly viewed as opening a fresh path to auditory science, and to the tools and techniques for diagnosing the functional status of the cochlea. But in terms of fundamental understanding, a key paper remains that of Thomas Gold more than half a century ago (Gold 1948). Still cited widely today, this paper deals with the basic question of how the cochlea works to analyse sound into its component frequencies. Two prominent theories—sympathetic resonance, proposed by Hermann Helmholtz (1885), and travelling waves, proposed by Georg von Békésy (1960)—need to be distinguished (Figure 1). In a nutshell, are there tiny, independently tuned elements in the cochlea, like the discrete strings of a piano, that are set into sympathetic vibration by incoming sound (Helmholtz), or is the continuously graded sensing surface of the cochlea hydrodynamically coupled so that, like flicking a rope, motion of the eardrum and middle ear bones causes a travelling wave to sweep from one end towards the other (von Békésy)?

**Figure 1:**
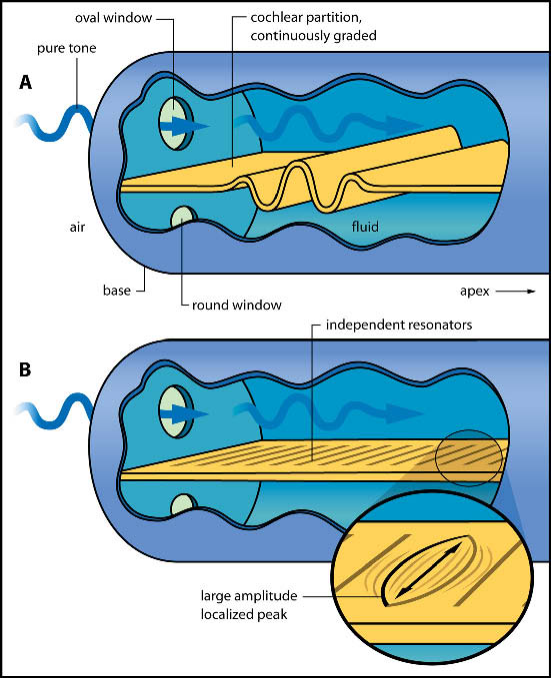
Two Views of Cochlear Mechanics The cochlea, shown uncoiled, is filled with liquid. In the accepted travelling wave picture (A), the partition vibrates up and down like a flicked rope, and a wave of displacement sweeps from base (high frequencies) to apex (low frequencies). Where the wave broadly peaks depends on frequency. An alternative resonance view (B) is that independent elements on the partition can vibrate side to side in sympathy with incoming sound. It remains open whether the resonant elements are set off by a travelling wave (giving a hybrid picture) or directly by sound pressure in the liquid (resonance alone).

The first option, sympathetic resonance, has the advantage of allowing vanishingly small energies to build up, cycle by cycle, into an appreciable motion—like boosting a child on a swing. The second, travelling wave, has the weight of von Békésy's extensive experiments behind it. At the same time, one of the drawbacks of the travelling wave theory is the difficulty of accounting for the ear's exquisite fine tuning: trained musicians can easily detect tuning differences of less than 0.2%. Even von Békésy himself notes, on page 404 of his classic book, that ‘the resonance theory of hearing is probably the most elegant of all theories of hearing’.

Gold's work, done in collaboration with RJ Pumphrey (Gold and Pumphrey 1948), was the first to consider that the ear cannot act passively, as both Helmholtz and von Békésy had thought, but must be an active detector. Gold was a physicist who had done wartime work on radar, and he brought his signal-processing knowledge to bear on how the cochlea works. He knew that, to preserve signal-to-noise ratio, a signal had to be amplified before the detector, and that ‘surely nature can't be as stupid as to go and put a nerve fibre—that is a detector—right at the front end of the sensitivity of the system’. He therefore proposed that the ear operated like a regenerative receiver, much like some radio receivers of the time that used positive feedback to amplify a signal before it was detected. Regenerative receivers were simple—one could be built with a single vacuum tube—and they provided high sensitivity and narrow bandwidth. A drawback, however, was that, if provoked, the circuit could ‘take off’, producing an unwanted whistle. Gold connected this with the perception of ringing in the ear (tinnitus), and daringly suggested that if a microphone were put next to the ear, a corresponding sound might be picked up. He experimented, placing a microphone in his ear after inducing temporary tinnitus with overly loud sound. The technology wasn't up to the job—in 1948 microphones weren't sensitive enough—and the experiment, sadly, failed.

Gold's pioneering work is now acknowledged to be a harbinger of Kemp's discoveries. But there is one aspect of Gold's paper that is not so widely considered: Gold's experiments led him to favour a resonance theory of hearing. In fact, the abstract of his 1948 paper declares that ‘previous theories of hearing are considered, and it is shown that only the resonance theory of Helmholtz… is consistent with observation’.

Gold and Pumphrey did psychophysical experiments in which hearing thresholds were determined for listeners first for continuous pure tones and then for increasingly briefer stimuli of the same frequency. Gold and Pumphrey showed that their results could only be accounted for by considering the cochlea as a set of resonators, each of which responds to a narrow frequency range.

In a second neat experiment, listeners had to detect differences between the sound of repetitive tone pips (series one) and those same stimuli but with the phase of every second pip inverted (series two, in which compressions replaced rarefactions and vice versa). Out-of-phase pips should counteract the action of in-phase pips and, following the child-on-swing analogy, rapidly bring swinging to a halt. Therefore, the argument goes, the two series should sound different. By increasing the silent interval between pips until the difference disappeared, the experimenters could infer how long the vibrations (or swinging) appeared to persist and could then put a measure on the quality factor *(Q),* or narrowness in frequency range, of the presumed underlying resonance.

From the first experiment, Gold and Pumphrey derived values of *Q* of 32 to 300, meaning that the range of response was as little as 1/300-th of the imposed frequency—based on the picture of a broad travelling wave. The second experiment gave comparable results. However, their resonance interpretation has been dismissed because of a methodological flaw in the second experiment: the spectral signatures of the two series are not the same and provide additional cues. Nevertheless, it is not widely appreciated that the first experiment seems methodologically sound, and its results remain persuasive.

I think the resonance theory deserves reconsideration. The evidence of my ears tells me that the cochlea is very highly tuned, and an active resonance theory of some sort seems to provide the most satisfying explanation. Furthermore, as well as Gold's neglected experiment, we now know from studies of acoustic emissions that the relative bandwidth of spontaneously emitted sound from the cochlea can be 1/1000 of the emission's frequency, or less. My research, guided by Professors M. V. Srinivasan and N. H. Fletcher, has centred on finding an answer to that most fundamental question: if the cochlea is resonating, what are the resonant elements?

A point of inspiration for me is Gold's later discussion of cochlear function (Gold 1987)—some nine years after Kemp's discoveries had been made. Gold draws a striking analogy for the problem confronting the cochlea, whose resonant elements—whatever they are—sit immersed in fluid (the aqueous lymph that fills the organ). To make these elements resonate is difficult, says Gold, because they are damped by surrounding fluid, just like the strings of a piano submerged in water would be. He concludes that, to make ‘an underwater piano’ work, we would have to add sensors and actuators to every string so that once a string is sounded the damping is counteracted by positive feedback. ‘If we now supplied each string with a correctly designed feedback circuit,’ he surmises, ‘then the underwater piano would work again.’

My research is investigating what Gold's underwater piano strings might be. A suggestion put forward in a recent paper (Bell and Fletcher 2004) is that resonance might occur in the space between the cochlea's geometrically arranged rows of outer hair cells. These cells are both effectors (they change length when stimulated) and sensors (their stereocilia detect minute displacements), so a positive feedback network can form that sets up resonance between one row of cells and its neighbour. The key is to transmit the feedback with the correct phase delay, and the new paper describes how this can be done using ‘squirting waves’ in the gap occupied by the outer hair cell stereocilia. The paper suggests that the outer hair cells create a standing wave resonance, from which energy is delivered to inner hair cells (where neural transduction takes place). In this way, the input signal is amplified before it is detected—an active system functioning just like Gold's regenerative receiver.

With a prime candidate in place for the resonating elements, this should, I think, prompt us to re-evaluate resonance theories of hearing, which were first put forward by the ancient Greeks and which, irrepressibly, keep resurfacing. The best-known resonance theory was that formulated by Helmholtz, but at that time no satisfactory resonating elements could be identified, and it lapsed until Gold's attempt to revive it. There are other difficulties in reviving a resonance theory of hearing, but I think they can be overcome.

If there really are resonant elements in the ear, the outstanding question would be, how are they stimulated? It is conceivable that motion of the conventional travelling wave sets them off, in which case we have an interesting hybrid of travelling wave and resonance. The other possibility, which I favour, is that outer hair cells are stimulated by the fast pressure wave that sweeps through all of the cochlear fluid at the speed of sound in water (1,500 m/s). If that is the case, and outer hair cells are primarily pressure sensors, not displacement detectors, then the ear is a fully resonant, pressure-driven system. New life, perhaps, to that old resonance idea.

## References

[pbio-0020337-Bell1] Bell A, Fletcher NH (2004). The cochlear amplifier as a standing wave: ‘Squirting’ waves between rows of outer hair cells?. J Acoust Soc Am.

[pbio-0020337-Davis1] Davis H (1983). An active process in cochlear mechanics. Hear Res.

[pbio-0020337-Gold1] Gold T (1948). Hearing. II. The physical basis of the action of the cochlea. Proc R Soc Lond B Biol Sci.

[pbio-0020337-Gold2] Gold T, Messel H (1987). The theory of hearing. Highlights in science.

[pbio-0020337-Gold3] Gold T, Pumphrey RJ (1948). Hearing. I. The cochlea as a frequency analyzer. Proc R Soc Lond B Biol Sci.

[pbio-0020337-Helmholtz1] Helmholtz HLF (1885). On the sensations of tone as a physiological basis for the theory of music.

[pbio-0020337-Kemp1] Kemp DT (1978). Stimulated acoustic emissions from within the human auditory system. J Acoust Soc Am.

[pbio-0020337-vonBekesy1] von Békésy G (1960). Experiments in hearing.

